# Leveraging Contingency Management to Encourage Online Brain Training Among Economically Disadvantaged Seniors: A Pilot Study

**DOI:** 10.7759/cureus.87921

**Published:** 2025-07-14

**Authors:** Jayla Hsiung, Lucy Nalepa, Qige Pan, Delya Chiu, Karan Patel, Jeffrey Chao

**Affiliations:** 1 Pre-Med/Applied Math, Brown University, Providence, USA; 2 Senior Connection, Highland Park High School, Highland Park, USA; 3 Neurology, Vanderbilt University Medical Center, Nashville, USA; 4 Internal Medicine, Kaiser Permanente, San Fransisco, USA

**Keywords:** brain training, cognitive health, cognitive training, contingency management, digital literacy, economically disadvantaged seniors, elderly individuals, financial incentive, senior wellness, technology access

## Abstract

This pilot study evaluated the feasibility of using contingency management (CM) to encourage economically disadvantaged seniors to participate in an online brain training program. Of 100 residents at a Housing Authority apartment complex, 11 initially enrolled, incentivized by monetary rewards. Despite multiple follow-up events, only 2% continued to engage regularly in weekly exercises. Participation was limited by several barriers, including confusing flyer language, limited access to technology, and low digital literacy. Additionally, non-CM costs, such as providing technical resources and on-site support, were found to be four times higher than CM-related expenses. These findings suggest that while CM is a promising incentive, substantial additional investments are needed to sustain engagement. With a modest 2% sustained participation rate, the study highlights significant socioeconomic and technological challenges. However, if these barriers are addressed through integrated support systems and digital infrastructure investments, CM-based interventions could potentially reach and benefit hundreds of thousands of the estimated 6-8 million economically disadvantaged older adults nationwide. Overall, the findings underscore the need for comprehensive interventions that combine incentives, access to technology, and consistent technical support to promote cognitive health in underserved aging populations.

## Introduction

Our country’s growing aging population has amplified concerns about age-related cognitive decline, particularly among economically disadvantaged seniors with limited access to healthcare and support services. Multidomain interventions, combining diet, exercise, and cognitively stimulating activities, have been shown to delay the onset and progression of cognitive deterioration in older adults [1,2]. Activities such as mahjong, tai chi, and volunteering have been linked to enhanced neural efficiency, improved quality of life, and reduced caregiver burden [2,3]. Moreover, cognitive training interventions have demonstrated lasting effectiveness, with improvements in targeted cognitive abilities equivalent to reversing 7-14 years of age-related decline in cognitively healthy older adults [4].

However, much of this research has focused on relatively affluent senior populations, highlighting the need to adapt interventions for low-income seniors who often face additional barriers, including limited Internet access, low digital literacy, and financial constraints [5,6].

Contingency management (CM), based on BF Skinner’s operant conditioning theory, offers a structured method to reinforce positive behaviors, such as cognitive training, through financial incentives [7,8]. While CM has shown promise in various health-related applications [9], its use in promoting cognitive health among underserved older adults remains underexplored. In theory, combining monetary rewards with online cognitive training should enhance engagement and promote sustained participation [9,10]. However, persistent digital disparities, including inadequate connectivity, lack of devices, and limited digital skills, underscore the need to pair incentives with strong infrastructural and technical support, particularly in public housing settings where resources are scarce [5,6]. To date, no published studies have piloted CM-based cognitive training in low-income senior housing environments, despite these communities facing elevated risks of cognitive decline and digital exclusion [5,6,11].

This pilot study aimed to assess the feasibility and identify the challenges of implementing an incentive-based cognitive training program in a low-income senior housing complex. We describe the recruitment strategies, technical infrastructure, and CM framework employed, highlighting how these components worked together to facilitate initial engagement and continued participation. By evaluating both quantitative outcomes (e.g., enrollment and retention rates) and qualitative observations (e.g., misunderstanding of incentives, lack of access to personal devices), this report offers insights into the feasibility, cost-effectiveness, and scalability of CM-supported brain training programs in underserved senior communities.

## Technical report

Context and setting

The study was conducted in a Housing Authority apartment building located in a small municipality in the mid-Atlantic region of the United States. The building consists of 100 single-bedroom units designated for low-income seniors, all of which were occupied during the project period. Prior to this initiative, the research team had established rapport with residents by hosting monthly social events, such as pizza parties and game nights, over a span of nearly two years. 

To be eligible for participation, residents were required to be fluent in English, as both the online platform and recruitment materials were available only in English. Participants also needed to be neurologically healthy, capable of comprehending the training content, and able to read and hear the module information presented. Specifically, participants were required to have sufficient visual and auditory ability to interact with training materials on a phone, tablet, or computer. These eligibility criteria were based on the requirements of the BrainHealth Project platform, which was designed and validated for use with cognitively intact, English-speaking individuals with adequate sensory function. We acknowledge that these criteria may introduce sampling limitations, especially in linguistically diverse or medically variable populations [12].

Implementation procedures

The BrainHealth Project, a free digital platform developed by the Center for BrainHealth at the University of Texas at Dallas, was utilized for this study [12]. The platform incorporates decades of cognitive research into a mobile- and web-based training application that offers strategic thinking exercises, cognitive assessments, and progress-tracking tools. Its structured approach, ease of online accessibility, and zero-cost model aligned well with the study's objective to explore low-barrier cognitive interventions for a high-need, underserved population [12].

**Figure 1:**
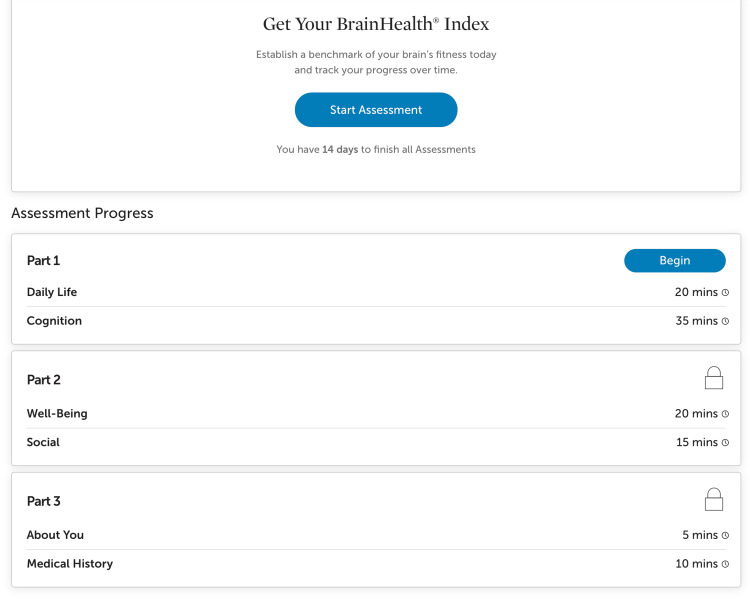
A screenshot of the BrainHealth Index assessment What users would see when they begin their BrainHealth Index assessment.

The BrainHealth Project web and mobile app provided several core features, including assessments such as the “BrainHealth Index,” which evaluates cognitive performance, emotional well-being, and social engagement (Figure 1).

**Figure 2:**
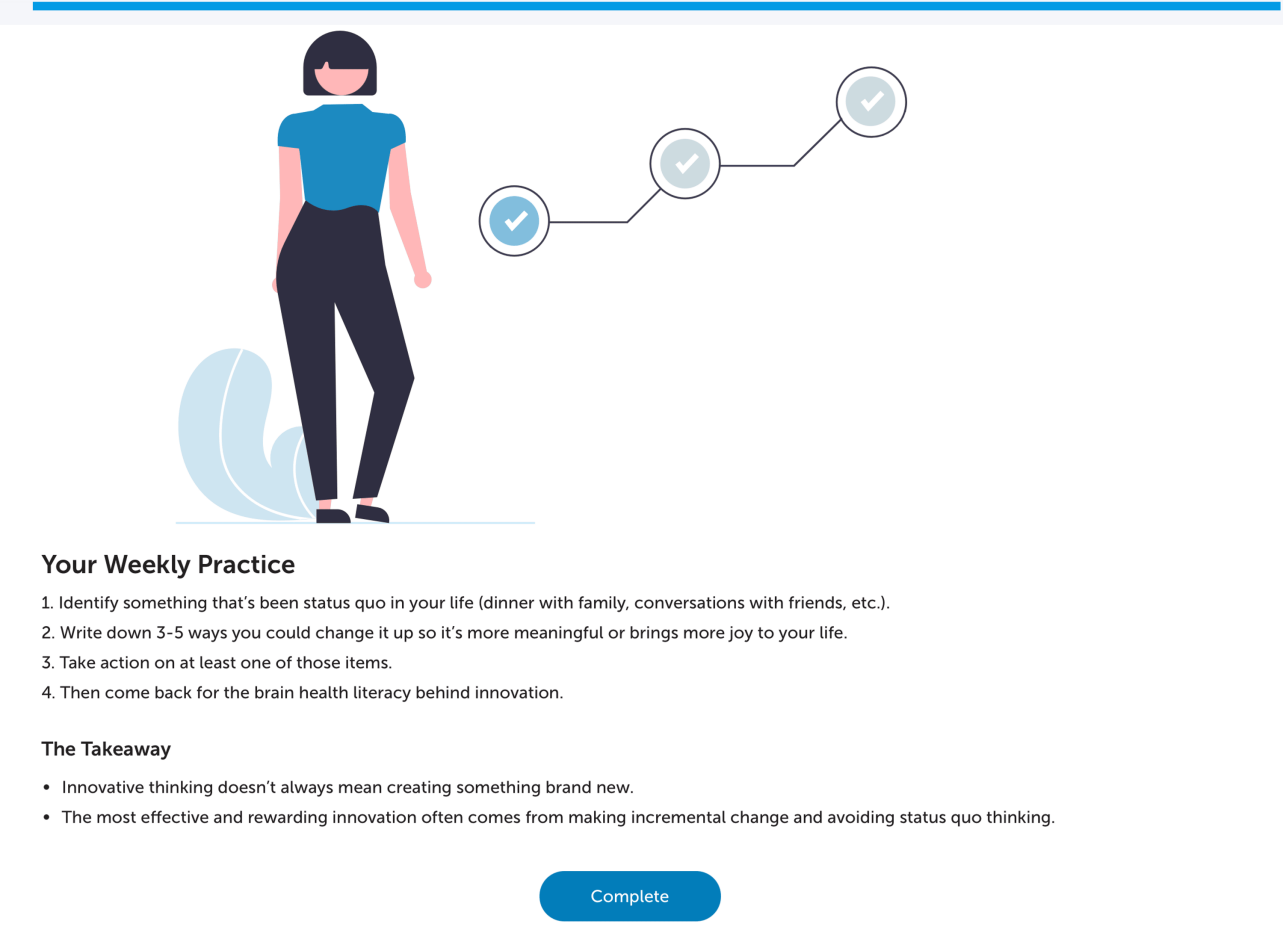
A screenshot of the innovative thinking brain exercise

The training modules consisted of self-paced online exercises based on “SMART Brain Training,” an evidence-based program designed to enhance brain performance and cognitive function across various age groups and populations [12]. SMART focuses on developing three core metacognitive strategies: strategic attention (filtering relevant information and minimizing distractions), integrated reasoning (abstracting key ideas and synthesizing information), and innovation (generating flexible, novel solutions, and perspectives in response to challenging content (Figure 2).

**Figure 3:**
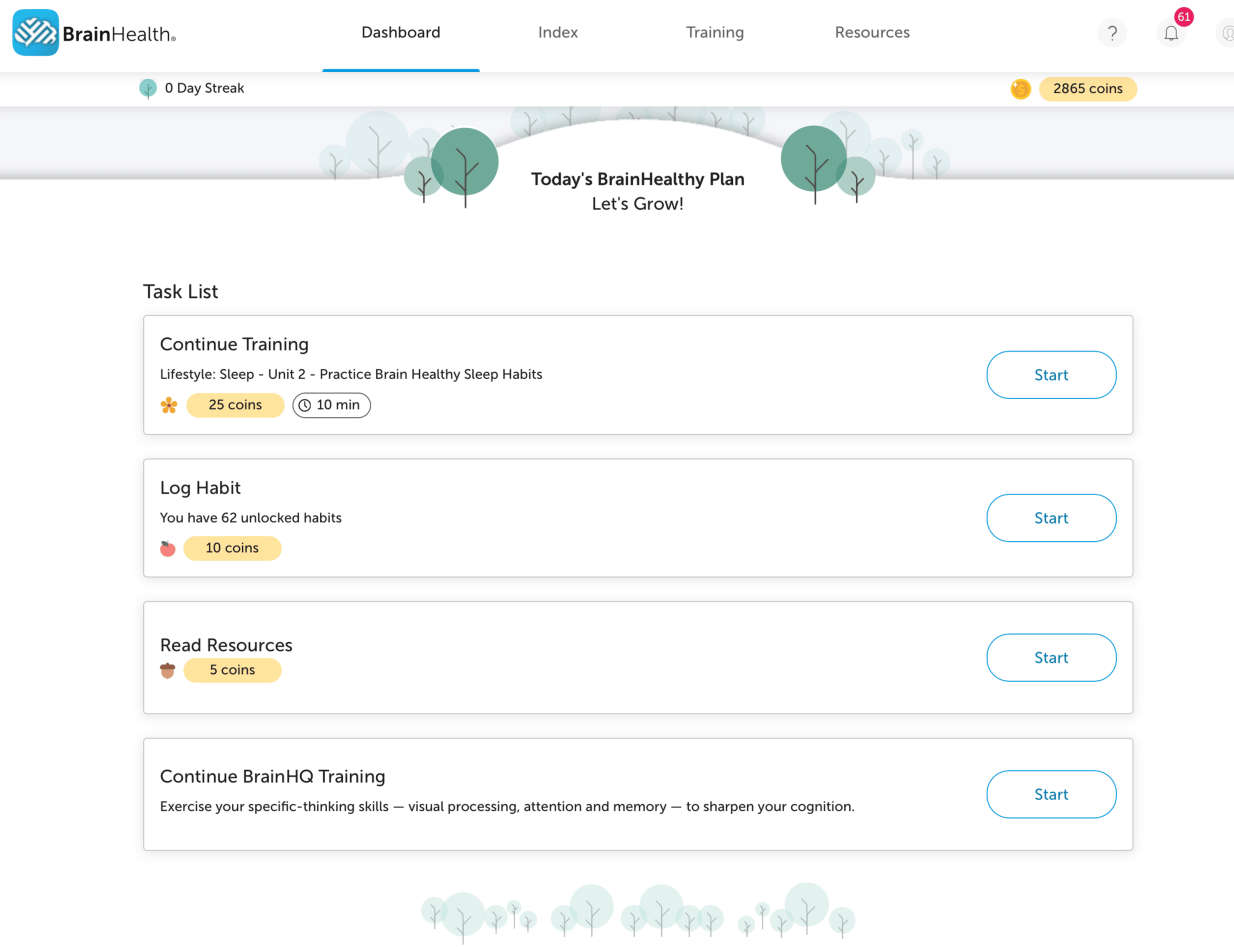
A screenshot of the BrainHealth training interactive dashboard

In a randomized pilot study, innovative cognition training resulted in measurable gains in innovative thinking among older adults, with cognitive improvements correlated with functional brain changes in neuronal networks associated with reasoning and creativity [13]. In addition, the app featured interactive dashboards that enable users to track their progress, view their brain training task list, monitor incremental changes in BrainHealth metrics over time, and access supplementary resources (Figure 3).

Optional in-app coaching prompts and automated reminders were available to all users. Because the app required only 5-10 minutes of daily engagement, it aligned well with the capabilities and routines of the older population. Importantly, the platform's no-cost structure reduced financial barriers, allowing the study to focus on whether a modest incentive through CM could help sustain participation among residents with limited resources [12].

The recruitment strategy involved distributing flyers to all 100 apartment units, announcing a registration event featuring a $10 sign-up incentive and free sandwiches. The research team facilitated an onsite registration session in the community room, where attendees could register in person and immediately receive the $10 reward. Subsequent follow-up events included verbal reminders during the initial event, additional flyers promoting refreshments such as cookies and hot cocoa, and announcements of increased incentives, raising the reward from $5 to $10 for completing exercises. Text message reminders were also sent to already registered participants, alerting them to upcoming opportunities to earn incentives through continued engagement.

The CM design included a $10 registration incentive upon successful sign-up for the training platform. A completion incentive for each follow-up session, initially set at $5 but increased to $10 during later follow-up events to boost participation. Participants received incentives in-person, immediately after demonstrating completion of the required brain training exercises.

To support residents without personal devices, the team installed a new laptop in the community room. Research staff provided basic demonstrations, including how to navigate the website and create user accounts, during follow-up events. Despite these efforts, some residents were confused by the wording on the flyers, with a few believing they needed to pay $10 rather than being paid. In response, the flyer language was revised in subsequent rounds to clarify this misunderstanding.

Observations and outcomes

**Figure 4:**
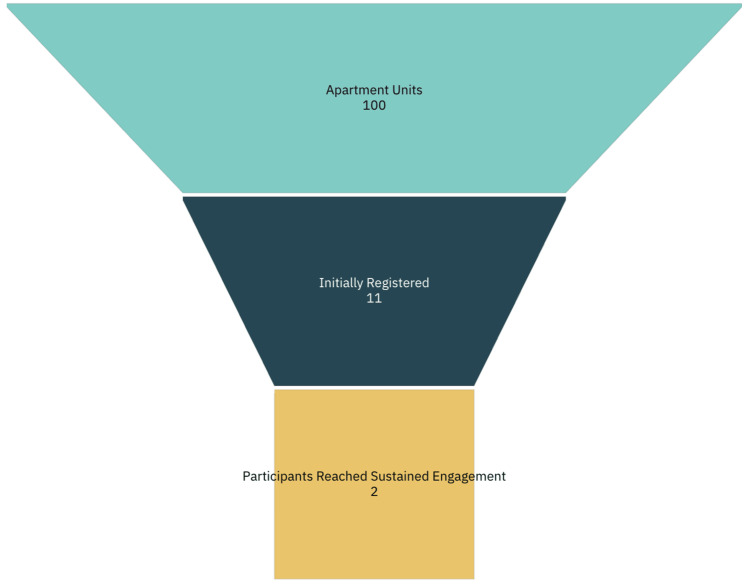
Funnel chart displaying participant recruitment and retention process

Initially, eight residents registered during the onsite event, with three additional registrations during follow-up sessions, resulting in a total enrollment of 11 participants, representing 10% of the building’s population. However, on average, only two residents (2%) attended follow-up events and earned incentives for completing the exercises. Most participants dropped out shortly after the registration phase. Figure 4 illustrates the recruitment and retention process, beginning with the broader target apartment population, narrowing to those approached and enrolled, and concluding with the small subset who achieved sustained engagement with the intervention.

Several barriers to participation emerged during the study. One significant issue was misinterpretation of the incentives, as some residents mistakenly believed they were required to pay $10 rather than receive it. In addition, a lack of personal technology hindered independent engagement, as several residents expressed interest but did not own a smartphone, tablet, or computer. Low digital literacy further compounded these challenges. Many residents did not have email accounts or basic Internet navigation skills, making it difficult to register, log in, and consistently access the brain training platform.

A breakdown of project expenses is shown in Table 1, illustrating that while CM incentive costs were modest, infrastructure and support expenses, such as purchasing a laptop and providing food, were four times higher than the direct CM-related costs. The $570 computer expense represents a one-time investment in a shared device placed in the community room to increase digital access. The total project cost was $965, including $570 for the community computer, $225 for food, $110 for registration incentives, and $60 for follow-up exercise completion incentives. Thus, CM-related costs totaled $170, while non-CM costs accounted for $795.

**Table 1:** Breakdown of expenses

Item	Cost ($)
Food	225
Laptop	570
Registration incentives (CM)	110
Follow-up incentives (CM)	60
CM-associated costs	170
Non-CM costs	795
Total cost	965

**Figure 5:**
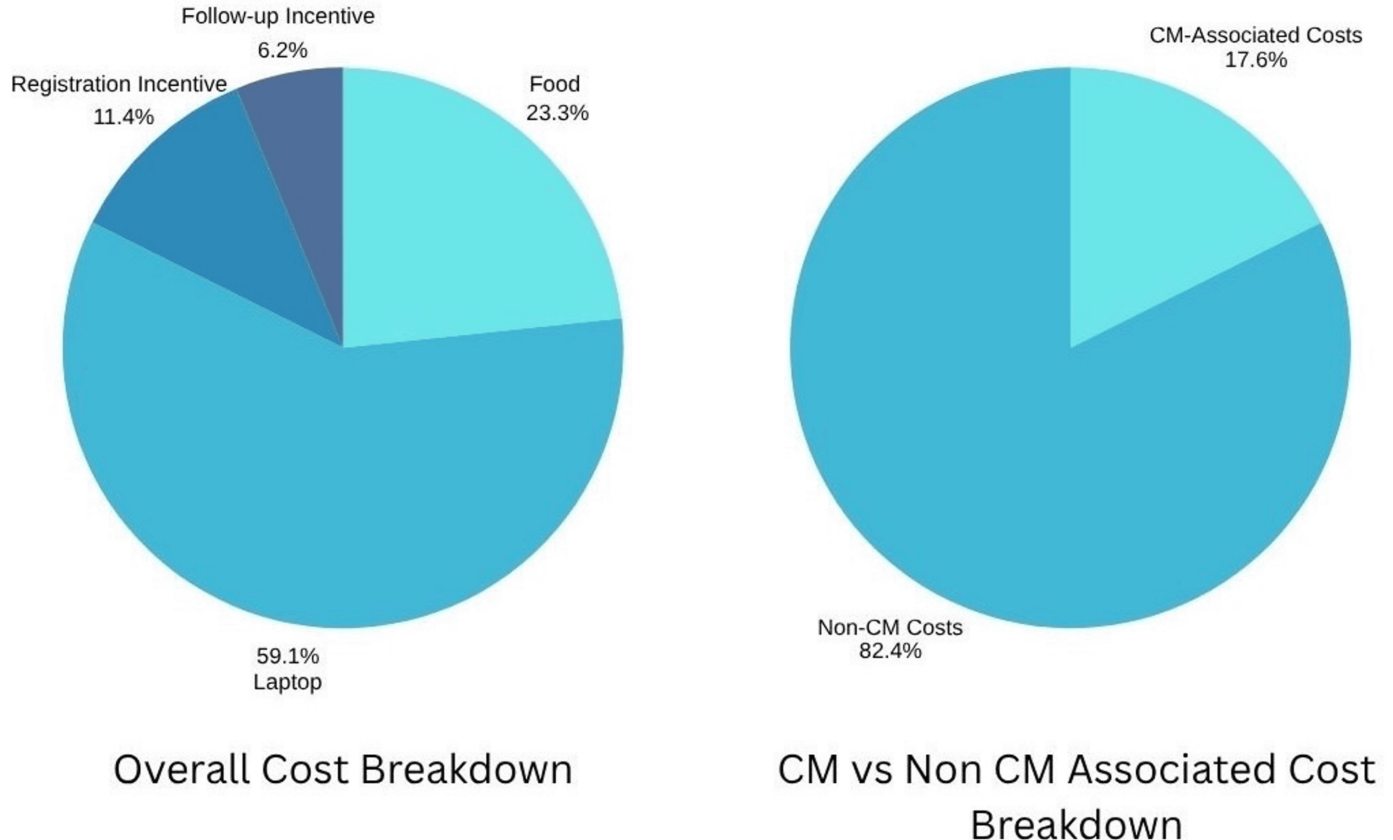
Pie chart representation of project expenses detailed in Table 1

Figure 5 provides a graphical representation of the project expenses outlined in Table 1.

## Discussion

The findings of this study highlight both the potential and the challenges of using CM to promote cognitive training among economically disadvantaged seniors. Although the project achieved only a modest 2% sustained engagement rate, this outcome should not diminish the broader promise of CM-based interventions. Instead, it underscores the substantial socioeconomic obstacles, including financial limitations, low digital literacy, and limited access to technology, which must be addressed for cognitive programs to succeed in underserved senior populations. These findings align with global public health goals, such as those outlined by the World Health Organization, which emphasize the importance of creating supportive, accessible environments tailored to older adults with limited resources [14].

Despite these barriers, prior research confirms that cognitive training, particularly when integrated with other health-promoting interventions (e.g., diet and exercise), can significantly enhance the well-being of older adults [1,2]. Multidomain interventions have been shown to slow cognitive decline in elderly populations [1], while culturally tailored, home-based programs have improved quality of life and reduced caregiver burden [15,16]. Moreover, complex cognitive training has been associated with enhanced resting-state brain activity and functional connectivity, potentially boosting neurovascular function in healthy seniors [17]. These findings affirm the value of pursuing cognitive interventions in low-income senior communities.

In our study, we assumed that most participants would continue engaging with the online training modules after registration. However, only a small fraction (20% of those registered, or 2% of the entire building population) remained engaged. Many residents required ongoing support to navigate the platform, underscoring the importance of structured, hands-on demonstrations. These difficulties reflect the broader “digital divide,” in which older, low-income adults often lack both the tools and skills to use digital technologies effectively [5,18].

To improve adherence, researchers have explored additional behavioral strategies beyond financial incentives, such as gamification, habit anchoring, and social accountability. Gamified elements, including visual appeal, goal-setting, and feedback, can increase engagement and enjoyment, but do not always guarantee sustained use, especially if tasks are frustrating or less enjoyable than control activities [19]. Habit anchoring, often implemented through “if-then” plans, links new behaviors with existing routines to encourage automaticity, though findings on its effectiveness are mixed [20]. Social accountability, where performance is shared or interdependent with others, has shown promise in boosting motivation and persistence. For instance, interventions where financial rewards are contingent on a partner's performance can lead to significantly higher inter-partner correlations in activity completion and greater persistence after incentives are withdrawn, suggesting that social interdependence can amplify motivation [10]. However, disengagement by one member can negatively affect others’ adherence, making this approach a double-edged sword [10].

A growing body of evidence confirms that user-friendly digital design and ongoing technical support are essential for sustained engagement. A scoping review by Wilson et al. identified intuitive interfaces and consistent assistance as key facilitators for e-health engagement among older adults [21]. Similarly, Stamate et al. emphasized user-centered design and digital training as critical to improving technology adoption in aging populations [22]. National organizations like AARP have taken proactive steps through programs such as Digital Skills Ready@50+™, which offers free training in essential tech skills to help seniors remain socially and cognitively engaged [23]. Partnerships with such initiatives could enhance digital capacity, social support, and long-term adherence in low-income communities. Furthermore, senior center-based interventions consistently underscore the value of on-site technical support in maintaining participation [24]. Simply put, getting started is not enough; ongoing support is essential.

Our cost analysis further illuminated the practical limitations of CM implementation. While direct CM incentives accounted for only a small portion of overall costs, infrastructure and support expenses, such as laptops and food, were substantially higher. This is consistent with findings from other CM programs (albeit in different health contexts), which have shown that administrative and infrastructure costs often outpace incentive payments [25]. These findings suggest that ignoring technology-related costs may lead to an incomplete understanding of what is truly required to implement CM effectively in underserved communities. National programs such as the Digital Equity Act, administered by the National Telecommunications and Information Administration (NTIA), now recognize the importance of investing in broadband access and digital literacy as critical to addressing disparities among older adults [26].

Psychological and social factors may also influence older adults' engagement with cognitively demanding tasks. Social comparison theory suggests that individuals assess their performance in relation to peers, which can either motivate or discourage participation [27,28]. Negative self-comparisons may lower self-efficacy and lead to disengagement [29,30], while positive evaluations can enhance persistence [31]. Building “brain health communities” where participants share progress, experiences, and encouragement may foster a more supportive, motivating environment. The Centers for Disease Control and Prevention (CDC) has emphasized the effectiveness of group-based interventions for older adults, which can reduce social isolation, enhance psychological well-being, and support health-promoting behaviors [32]. By providing opportunities for regular social interactions, group-based settings encourage older adults to form meaningful connections, engage in mutual encouragement, and establish shared goals. These social dynamics not only mitigate feelings of loneliness but also foster a supportive atmosphere that enhances both psychological well-being and long-term adherence to health-promoting activities.

Given the rising number of older adults living at or below the poverty line [6,33], scaling CM-based interventions holds important public health potential. However, broad expansion would be premature without addressing the critical socioeconomic and technological barriers currently limiting engagement. Rather than projecting national impact figures, our findings suggest that scaling success hinges on meeting key preconditions, including subsidized broadband, device access, and targeted digital literacy support [26]. As our cost analysis revealed, non-CM investments may far exceed CM-related costs, but are essential for long-term sustainability in high-need populations.

This study has several limitations that warrant consideration. First, the eligibility criteria requiring English fluency and neurological health likely introduced sampling bias, limiting the generalizability of findings to linguistically diverse populations or seniors with pre-existing cognitive impairments. By excluding non-English speakers and individuals with neurological conditions, the results may not reflect the experiences of more medically or culturally heterogeneous groups. Second, the single-site design and small sample size constrain external validity, as the pilot was conducted in one low-income senior housing community with unique demographic and infrastructural characteristics. To confirm generalizability, broader implementation would require validation across diverse settings (e.g., rural areas or non-English-speaking populations). Third, as a pilot study designed to test feasibility, the protocol did not include a control group, which prevents us from making causal claims about the efficacy of CM. Lastly, our study did not include a qualitative component, such as participant interviews, to analyze the specific behavioral reasons for disengagement. 

To address these limitations, a future randomized controlled trial (RCT) is the necessary next step to determine causality. Future research should also employ a qualitative component, such as participant interviews, to better capture the reasons behind the user engagement or disengagement. Additionally, future studies should prioritize multi-site trials with expanded eligibility criteria and multilingual platforms to better represent underserved seniors' socioeconomic and medical diversity. Integrating technological support and digital education directly into cognitive training programs could clarify whether such measures improve long-term adherence. Exploring alternative incentive designs, such as tiered or group-based rewards, may also prove valuable, particularly in low-resource settings. Further inquiry should examine how peer support, mental health factors, and partnerships with public housing authorities, local governments, and community-based organizations enhance engagement and intervention effectiveness. By combining practical supports, evidence-based motivational strategies, and inclusive technological infrastructures, future programs could deliver meaningful cognitive health benefits to economically disadvantaged seniors.

## Conclusions

This pilot study demonstrates that CM can effectively generate initial interest in online cognitive training among economically disadvantaged seniors. However, sustaining long-term participation requires hybrid interventions that integrate financial incentives with robust technological and social infrastructure. The modest 2% sustained engagement rate underscores the impact of socioeconomic barriers, such as limited digital literacy and/or access to personal technology, which calls for holistic solutions. While CM incentives are critical for motivating enrollment, they are insufficient alone; success hinges on pairing rewards with accessible digital tools, hands-on training, and community-driven support. Cognitive training interventions remain promising for improving quality of life and slowing cognitive decline in older adults. To realize this potential, programs must adopt a hybrid model that combines financial incentives with investments in broadband access, affordable devices, and digital literacy education. Collaborative partnerships with public housing authorities, local governments, and advocacy organizations (e.g., AARP) can amplify reach and sustainability. The potential for national impact is significant, but our findings confirm that it is entirely contingent on policymakers and practitioners first prioritizing infrastructure support alongside motivational strategies such as CM.

Future research must not only optimize hybrid interventions but also employ more rigorous methodologies to build on the lessons from this pilot. This includes designing randomized controlled trials (RCTs) to definitively establish causality, as well as incorporating qualitative methods to better understand the user experience. Within these frameworks, researchers should continue to test specific strategies like tiered incentive structures and evaluate how peer support groups enhance adherence. By embedding CM within a framework of technological accessibility, community engagement, and rigorous scientific evaluation, we can help turn online registrations into lasting cognitive health gains for economically disadvantaged seniors.
